# Impact of Endocytosis and Lysosomal Acidification on the Toxicity of Copper Oxide Nano- and Microsized Particles: Uptake and Gene Expression Related to Oxidative Stress and the DNA Damage Response

**DOI:** 10.3390/nano10040679

**Published:** 2020-04-03

**Authors:** Bettina Maria Strauch, Wera Hubele, Andrea Hartwig

**Affiliations:** Department of Food Chemistry and Toxicology, Institute of Applied Biosciences (IAB), Karlsruhe Institute of Technology (KIT), 76131 Karlsruhe, Germany; bettina_strauch@gmx.de (B.M.S.); W.Hubele@gmx.de (W.H.)

**Keywords:** copper oxide nanoparticles, genomic stability, gene expression profiling, high-throughput RT-qPCR, endocytosis, cellular copper uptake, lysosomal acidification, Trojan horse-type mechanism

## Abstract

The toxicity of the copper oxide nanoparticles (CuO NP) has been attributed to the so-called “Trojan horse”-type mechanism, relying on the particle uptake and extensive intracellular release of copper ions, due to acidic pH in the lysosomes. Nevertheless, a clear distinction between extra- and intracellular-mediated effects is still missing. Therefore, the impact of the endocytosis inhibitor hydroxy-dynasore (OH-dyn), as well as bafilomycin A1 (bafA1), inhibiting the vacuolar type H^+^-ATPase (V-ATPase), on the cellular toxicity of nano- and microsized CuO particles, was investigated in BEAS 2 B cells. Selected endpoints were cytotoxicity, copper uptake, glutathione (GSH) levels, and the transcriptional DNA damage and (oxidative) stress response using the high-throughput reverse transcription quantitative polymerase chain reaction (RT-qPCR). OH-dyn markedly reduced intracellular copper accumulation in the cases of CuO NP and CuO MP; the modulation of gene expression, induced by both particle types affecting especially *HMOX1*, *HSPA1A*, *MT1X*, *SCL30A1*, *IL8* and *GADD45A*, were completely abolished. BafA1 lowered the intracellular copper concentration in case of CuO NP and strongly reduced transcriptional changes, while any CuO MP-mediated effects were not affected by bafA1. In conclusion, the toxicity of CuO NP depended almost exclusively upon dynamin-dependent endocytosis and the intracellular release of redox-active copper ions due to lysosomal acidification, while particle interactions with cellular membranes appeared to be not relevant.

## 1. Introduction

Copper oxide nanoparticles (CuO NP) are increasingly applied as catalysts, additives in plastics, or as antimicrobial agents [1]. Thus, a detailed toxicological knowledge is important as a prerequisite to ensure an appropriate risk assessment due to enhanced exposure and the potential risk of adverse health effects, especially with respect to occupational exposure. Pronounced cytotoxic and genotoxic effects of CuO NP, compared to other metal-based nanoparticles, bigger counterparts of the same chemical composition, as well as water soluble copper compounds, was repeatedly demonstrated in various cell culture models. As an underlying reason, a CuO NP-induced intracellular copper overload was reported and proposed to be most decisive for adverse reactions [2,3,4,5,6,7,8], named as the “Trojan horse” mechanism [2,9]. 

According to this theory, the particles are taken up via endocytic pathways, delivering large amounts of CuO within vesicles, which are further processed into acidic lysosomes, leading to an enhanced dissolution of the particles, and resulting in an extensive intracellular overload with copper ions. Thus, strict homeostatic control of copper uptake via transporters like high-affinity copper transporter 1 (hCTR1) is bypassed, and redox-active copper ions provoke the oxidative stress induced by hydroxyl radicals via Fenton-type reactions, causing oxidative damage to DNA, lipids and proteins [10]. Furthermore, the high thiol affinity of copper ions turns redox-sensitive cysteines, present for example in zinc-binding protein structures, but also in the active sites of several enzymes, into important targets of copper toxicity. Accordingly, the modulation of zinc-binding structures which are present in several DNA repair and tumor suppressor proteins, but also in redox-regulated transcription factors, may lead to an interference with the DNA damage response system, either directly or indirectly via ROS as second messengers; this may further contribute to the toxicity of CuO NP [11,12]. The modulation of gene expression by CuO NP was investigated in detail in a previous study of our working group in adenocarcinoma A549 and BEAS-2B lung cells [8]. Gene expression profiling was performed by a high-throughput reverse transcription quantitative polymerase chain reaction (RT-qPCR) technique, quantifying the impact of 96 samples in parallel on the expression of 95 genes crucial for maintaining genomic stability. Selected genes were related to metal homeostasis, an (oxidative) stress response as well as DNA repair, cell cycle regulation and apoptosis [13]. A pronounced dose-dependent impact on copper uptake, an oxidative stress response, DNA damage response and apoptosis-associated genes, was observed in case of the CuO NP, concomitant with a distinct copper overload in the cytoplasm, and especially the nucleus. Far weaker effects were observed after treatment with micro-sized CuO particles, or with water-soluble copper chloride. Furthermore, along with the marked activation of the DNA damage response on the transcriptional level, concentration-dependent cytotoxicity, the induction of oxidative stress, cell cycle arrest, and apoptosis, were observed by CuO NP on the functional level in BEAS-2B cells [8]. 

Following up on these observations, different aspects still need to be clarified. Even though the observations may well be explained by a Trojan horse-type mechanism described above, a clear link, as well as the exclusion of other direct cellular interactions of the NP, are still missing. Supporting evidence is provided by the demonstration of CuO NP uptake via different endocytic pathways [14,15,16,17,18]. Nevertheless, the distinction between the potential effects induced by the particles extracellularly, e.g., via interactions with membrane receptors, and effects arising due to intracellular copper ion release in acidic lysosomes, remains to be established. 

Therefore, within the present study, the impact of the dynamin-dependent endocytosis inhibitor hydroxy-dynasore (OH-dyn) [19,20], and the specific inhibitor of the vacuolar type H^+^-ATPase (V-ATPase) bafilomycin A1 (bafA1) (which prevents lysosomal acidification [21,22]), on the cellular copper uptake, cytotoxicity, and especially the modulation of gene expression by CuO NP, was investigated. Since the lung is the major target of CuO NP toxicity, experiments were performed in epithelial bronchial non-tumorigenic BEAS-2B cells, widely used for mechanistic in-vitro studies associated with lung toxicity, including lung carcinogenesis, and well suited for NP-induced gene expression analyses [8]. The effects of CuO NP were compared to CuO MP and water-soluble CuCl_2_ to distinguish between the particle, nanoparticle or copper ion-mediated effects, and to identify potential unspecific or side-effects of the inhibitor substances. 

Our results confirmed a relevant participation of dynamin-dependent endocytosis in the uptake of CuO NP and CuO MP by the use of OH-dyn. Furthermore, bafA1 was effective in preventing CuO NP-induced cytotoxicity and glutathione (GSH) depletion, coincident with decreased intracellular copper accumulation assumed to result from the stalled uptake of CuO NP, and thus diminishing the release of redox-reactive copper ions in less acidic lysosomes. The gene expression analyses confirmed that the CuO NP-mediated modulation of gene expression greatly depends on both endocytosis and lysosomal acidification, and consequently on intracellular copper ion release. 

## 2. Materials and Methods 

### 2.1. Materials

Chemicals, including agarose, salts, glycerol, leupeptine, phenylmethanesulfonyl-fluoride (PMSF), bovine serum albumin, acids, snap-on lid glasses and stirring bars, were obtained from Carl Roth GmbH (Karlsruhe, Germany). CuO NP (#544868, Lot #MKAA0633), CuO MP (#208841, Lot #MKAA1788) and copper chloride were purchased from Sigma-Aldrich Chemie GmbH (Steinheim, Germany). All PCR consumables, including PCR tubes, strips and reaction tubes and tubules, as well as cell culture dishes and flasks, were obtained from Sarstedt (Nuembrecht, Germany). The primer pairs were synthesized by Eurofins (Ebersberg, Germany) or Fluidigm (San Francisco, CA, USA). OH-dyn, Dulbecco’s modified Eagle’s medium (DMEM), dimethyl sulfoxide (DMSO), trypsin, amphotericin B, trypsin inhibitor from glycine max (soybean) (SBTI) and penicillin-streptomycin solutions are products of Sigma-Aldrich. Fetal calf serum (FCS) and LHC-9 media are products of Invitrogen GmbH (Darmstadt, Germany). BafA1 was purchased from Santa Cruz Biotechnology (Heidelberg, Germany). Human fibronectin was obtained from Biopur (Reinach, Switzerland) and collagen from Roche (Mannheim, Germany). DNA suspension buffer, PCR-certified water and TE buffer were obtained from Teknova (Hollister, CA, USA). 2X Assay Loading Reagent and 20X DNA Binding Dye Sample Loading Reagent were purchased from Fluidigm (San Francisco, CA, USA). Bio-Rad (Munich, Germany) provided the 2X SsoFastTM EvaGreen^®^ Supermix with Low ROX and the 2X SYBR Green Supermix. The 2X TaqMan^®^ PreAmp Master Mix was obtained from Applied Biosystems (Darmstadt, Germany) and the exonuclease I from New England Biolabs (Frankfurt am Main, Germany). 

### 2.2. Particle and CuCl_2_ Incubation Suspensions and Dilutions

Fresh CuO NP and CuO MP suspensions, as well as CuCl_2_ dilutions, were prepared for each experiment. Particles, received as dry powder, were aliquoted by weighing into 1.5 mL polystyrene reaction tubes. Stock solutions of 1 mg/mL CuO were prepared in an endotoxin-free snap-on glass, containing a stirring bar in DMEM containing 10% FCS. Dilutions in the range of 5, 10, 20 and 50 µg/mL were prepared by adding aliquots of the stirring stock solution into snap-on lid glasses filled with adequate volumes of fresh medium. Stirring took place at 900 rpm and room temperature on a multiphase stirrer (Variomag Poly, Carl Roth GmbH, Karlsruhe, Germany). CuCl_2_ was dissolved in bi-distilled H_2_O (100 mM) and sterile-filtered. The dilution to 630 µM corresponding to 50 µg/mL CuO was prepared from the stock solution by dilution in an adequate amount of DMEM containing 10% FCS directly before incubation. Based on the copper content, 1 µg/mL CuO is equal to 0.2 µg/cm^2^ CuO and 12.6 µM Cu^2+^ in case of complete dissolution.

### 2.3. Cell Culture and Incubation

Human lung bronchial epithelial BEAS-2B cells (ATCC CRL-9609), immortalized with SV40 large T-antigen, were kindly provided by Dr. Carsten Weiss (Karlsruhe Institute of Technology, Karlsruhe, Germany). They were grown as monolayers in coated cell culture dishes (100 mm, 58 cm^2^, coated with 10 µg/mL human fibronectin, 30 µg/mL collagen and 10 µg/mL bovine serum albumin in phosphate-buffered saline (PBS)) in 12 mL LHC-9 medium containing 2.5 µg/mL amphotericin B. Cells were incubated at 37 °C in a humidified atmosphere of 5% CO_2_ in air. For all of these experiments, the cells were seeded at a density of 16,600 cells/cm^2^. After 48 h the supernatant was removed from the logarithmically growing cells, and replaced by the particle or CuCl_2_ incubation suspensions (0.2 mL/cm^2^) in DMEM containing 10% FCS, as described for the respective experiments. For co-incubation with the endocytosis inhibitor, BEAS-2B cells were pre-incubated with 100 µM OH-dyn for 30 min, and afterwards co-treated with CuO NP, CuO MP or CuCl_2_ and OH-dyn for 8 h. Co-incubation of the copper compounds with 100 nM bafA1 occurred for 24 h without pre-treatment. 

Since stock solutions of OH-dyn (20 mM) and bafA1 (50 µM) were prepared in DMSO, the same amount of DMSO was added to the respective controls without the inhibitor substances. 

### 2.4. Cell Number

Logarithmically growing BEAS-2B cells were incubated for 24 h with 10 µg/mL CuO NP, 50 µg/mL CuO MP or 630 µM CuCl_2_, with or without 100 nM bafA1 or 100 µM OH-dyn, respectively, trypsinized and collected in DMEM containing 10% FCS. Cell number was determined via Casy^®^ cell counter (OLS OMNI Life Science GmbH and Co. KG, Bremen, Germany). 

### 2.5. Gene Expression Analyses

0.5–1 × 10^6^ logarithmically growing BEAS-2B cells were treated with different concentrations of CuO NP, CuO MP or CuCl_2_, with or without 100 µM OH-dyn or 100 nM bafA1 for 8 h or 24 h, respectively, in DMEM containing 10% FCS. Subsequently, gene expression analyses via high-throughput RT-qPCR with Fluidigm dynamic arrays on the BioMarkTM System were performed, as described previously [13]. For normalization, five potential reference genes were available (*ACTB*, *B2M*, *GAPDH*, *GUSB* and *HPRT1*). Finally, potential alterations of the transcript levels of the target genes under investigation were displayed as fold change compared to the respective control group, by calculating relative quantities corresponding to the ΔΔCq method [23]. 

### 2.6. Cellular Copper Uptake 

Logarithmically growing BEAS-2B cells were treated with 10 µg/mL CuO NP, 50 µg/mL CuO MP or 630 µM CuCl_2_, with or without 100 µM OH-dyn or 100 nM bafA1, respectively, for 8 h or 24 h, respectively, in DMEM containing 10% FCS. The cells were trypsinized, collected in ice-cold PBS containing 10% FCS, and washed twice with PBS. Cell number and cell volume were determined via Casy^®^ cell counter. To quantify the intracellular copper levels in the soluble cell fractions, and thus to eliminate particles attached at the outer cell membrane, the cells were lysed in radioimmunoprecipitation assay (RIPA) buffer (0.01 M Tris pH 7.6, 0.15 M NaCl, 0.001 M ethylenediaminetetraacetic acid (EDTA), 1% (*v*/*v*) Trition-X 100, 1% (*v*/*v*) desoxycholic acid, sodium salt, 0.01% sodium dodecyl sulfate (SDS) 0.001 M phenylmethylsulfonyl fluoride (PMSF), 1 × protease-inhibitor mixture) for 30 min before centrifugation at 14,000 × g (1 h, 4 °C), thereby precipitating the cell membranes. The supernatant contained the soluble cell fraction of both the cytoplasm and nucleus, and its copper content was determined using GF-AAS (Perkin Elmer Atomic Absorption Spectrometer PinAAcle 900T, Rodgau, Germany). The solution was evaporated at 95 °C, incubated with ashing mixture, 65% HNO_3_/30% H_2_O_2_ (1/1), evaporated at 95 °C again, and resolved in water. Copper content was normalized to cell number and cell volume.

### 2.7. Quantification of Intracellular Glutathione

The quantification of intracellular glutathione levels was performed according to the method established by Tietze et al. [24]. Briefly, logarithmically growing BEAS-2B cells were incubated for 2 h with 10 µg/mL CuO NP, with or without 100 nM BafA1, respectively, in DMEM containing 10% FCS. The cells were trypsinized, collected in ice-cold PBS containing 10% FCS, washed with PBS, analyzed via Casy^®^ cell counter for cell number and cell volume and collected by centrifugation. 1 × 10^6^ cells were collected in KP buffer (0.1 M KH_2_PO_4_, 0.1 M K_2_HPO_4_, 1 mM EDTA, pH 7.4). Cells were lyzed by 2 freeze-and-thawing cycles followed by sonification, acidification with sulphosalicyclic acid and vortexing. Cells were centrifuged at 16,000 rpm for 20 min at 4 °C prior to measuring total GSH in the supernatant by reducing oxidized GSH content using GR enzyme (4 U/mL) and the reduced form of nicotinamide adenine dinucleotide phosphate (NADPH) (0.3 mM). GSH reacts with 5,5’-dithiobis-2-nitrobenzoic acid (DTNB) to form 2-nitro-5-thiobenzoate (TNB). The change in TNB absorbance was measured at 412 nm using a plate reader (Tecan). Data were compared to GSH standard calibration curves and normalized to cell volume.

### 2.8. Statistics

Differences between control and treated samples were analyzed by one-way analysis of variance (ANOVA), followed by Dunnett’s T post hoc test, and the differences between the different incubation conditions with and without OH-dyn or bafA1 were analysed by T-test, respectively.

## 3. Results

### 3.1. Particle Characteristics

Particle characteristics are summarized in Table 1. CuO NP and CuO MP were characterized using DLS with respect to size, scanning electron microscopy (SEM) for size and morphology, Brunauer–Emmett–Teller (BET) for surface area, zeta potential (ZP) for surface charge, inductively coupled plasma mass spectrometry (ICP-MS), Energy-dispersive X-ray spectroscopy (EDX) and oxygen analysis for purity and composition, as well as X-ray diffraction (XRD) for crystallinity in two previous studies of our working group. Additionally, the impact on pH in relevant media and solubility in different fluid models, like DMEM containing 10% FCS and lysosomal artificial fluid (ALF), was investigated. Briefly, both CuO particles were of high purity (>99.8%), of equal composition, were free of endotoxins, and did not alter the pH of the cell culture media. CuO NP were approximately spherical, displayed a narrow size range (20–200 nm, with a calculated average diameter of 55 nm based on the specific surface area, and 146 nm in cell culture medium containing 10% FCS derived from the DLS measurements), a surface area of 17.23 m^2^/g, and a ZP of −13.1 mV in DMEM/FCS [7]. About 90% of the particles were in the range < 100 nm (Hufnagel and Hartwig, unpublished results). CuO MP showed a size distribution of 500 nm–10 µm (1289 nm calculated average diameter, DLS analysis was not feasible because of fast sedimentation), and a surface area of 0.74 m^2^/g [7]. 

The solubilities of both particle types have been investigated in our previous study in several model fluids in a time-dependent manner, quantified by graphite furnace atomic absorption spectroscopy (GF-AAS) [7], including artificial alveolar fluid (AAF, pH = 7.4, composed as described by Stopford and coworkers [25]) and artificial lysosomal fluid (ALF; pH = 4.5, composed as described by Midander and coworkers [26]). In H_2_O and AAF, dissolution for both particles types was below 2.4%, with CuO NP releasing more copper ions than the CuO MP. Nevertheless, the dissolution of CuO NP was highly accelerated in a time-dependent manner in the cell culture medium (DMEM) supplemented with FCS, reaching 44% after 24 h. In contrast, copper ion release from the CuO MP remained low with 4% dissolved copper after 24 h incubation. The accelerated solubility of the CuO NP as compared to the CuO MP was even more pronounced in the acidic environment of ALF, where 68% of the CuO NP was already solubilized after 30 min, and after 2 h dissolution was almost complete. In contrast, CuO MP revealed only 10% dissolution after 4 h, and about 80% solubilization was reached only after 168 h [7]. 

### 3.2. Cytotoxicity

**Figure 1:**
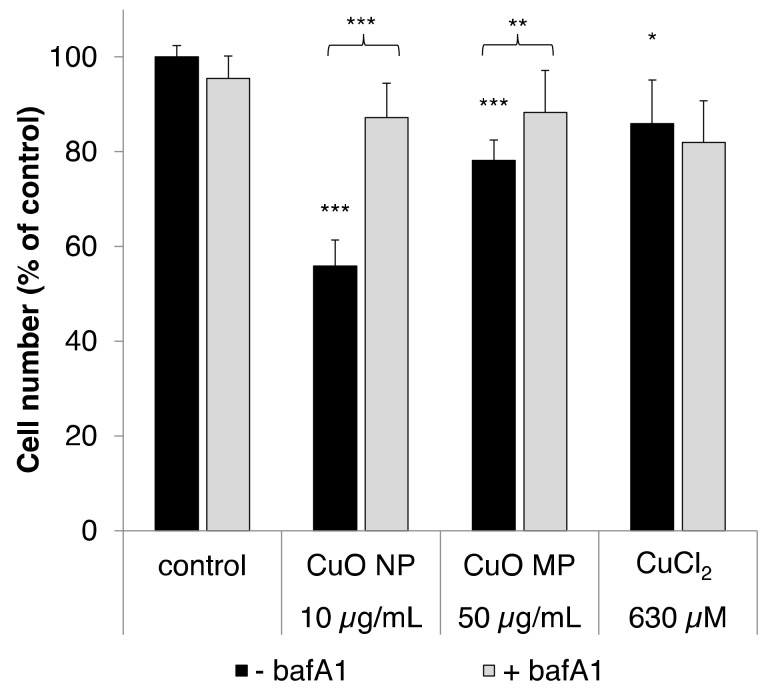
Cytotoxicity of copper oxide nanoparticles (CuO NP), copper oxide microparticles (CuO MP) and CuCl_2_ in the absence or presence of bafA1. Cytotoxicity was determined via cell number after 24 h treatment, with the different copper compounds co-treated with or without bafA1, respectively. Shown are mean values of five determinations derived from three independent experiments + standard deviation (SD). Statistically significant different from control or the respective copper compound treatment: * *p* ≤ 0.05, ** *p* ≤ 0.01, *** *p* ≤ 0.001 (T-test). Based on the copper content, 50 µg/mL CuO are equal to 630 µM Cu^2+^.

The cell number of BEAS-2B cells was determined as a measure of the cytotoxicity of CuO NP, CuO MP and CuCl_2_ after 24 h incubation (Figure 1). This approach was chosen due to the inability of BEAS 2B cells to form colonies, and a reported interference by copper-based particles and copper ions with dye-based toxicity assays [27]. A detailed investigation on the cytotoxicity of the copper compounds was performed in our previous study, revealing a pronounced concentration-dependent impact of CuO NP upon cell number, starting at 5 µg/mL and reaching 10% viability in case of 50 µg/mL, a moderate cytotoxic impact in case of CuO MP, and only weak cytotoxicity in case of CuCl_2_ [8]. Based on these findings, in the present study single concentrations of the respective compounds were selected and analyzed again for cytotoxicity; the results highly corresponded to the previous findings. Cytotoxicity studies were also performed with the dynamin-dependent endocytosis inhibitor hydroxy-dynasore (OH-dyn), as well as with the vacuolar H^+^-ATPase inhibitor bafA1, applying 100 µM or 100 nM, respectively, based on literature data ([19,20,21,22]). In the case of bafA1, applying 100 nM, almost no reduction in cell number was observed when following the usual protocol of 24 h used for co-incubation with the CuO particles (Figure 1). However, in case of 100 µM OH-dyn, the 24 h treatment resulted in only 60% residual viability compared to the control; therefore the incubation time was reduced to 8 h in this case in subsequent experiments. 

As shown in Figure 1, 10 µg/mL CuO NP exerted the most pronounced cytotoxicity, leading to a decrease of cell number to 56% compared to our control. In contrast, CuO MP and CuCl_2_ were far less cytotoxic, even though applied at 5-fold higher mass doses; thus, CuO MP decreased the cell number to 78% and CuCl_2_ to 86%. In the presence of the vacuolar H^+^-ATPase inhibitor bafA1, the cytotoxicity of the CuO particles was almost completely abolished, most strikingly in case of the CuO NP, but also in case of the CuO MP. Thus, the cell number was recovered to about 90% in both cases. However, no effect of bafA1 on the cytotoxicity of CuCl_2_ was observed, excluding unspecific interactions. 

### 3.3. Cellular Copper Uptake 

**Figure 2:**
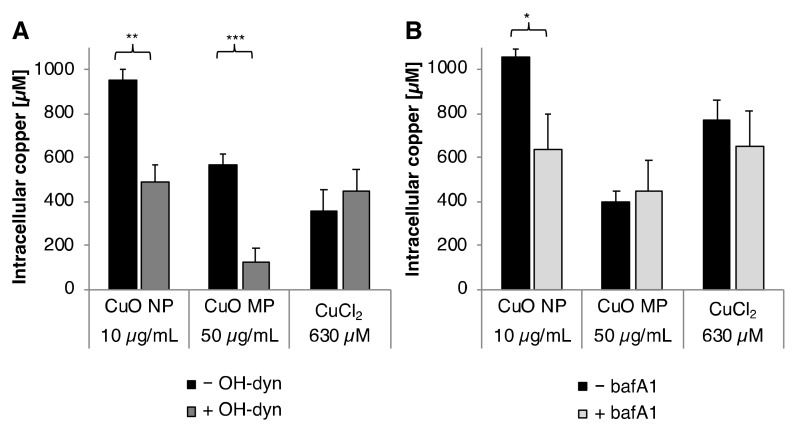
Cellular copper uptake after treatment with CuO NP, CuO MP or CuCl_2_ in the presence or absence of (**A**) OH-dyn and (**B**) bafA1. Copper content was determined in the soluble fraction of BEAS-2B cells after 8 h treatment, with the different copper compounds co-treated with or without OH-dyn, or after 24 h co-treated with or without bafA1, respectively, via GF-AAS. Shown are the mean values of three independent experiments + SD. Statistically significant different from the respective copper compound treatment: * *p* ≤ 0.05, ** *p* ≤ 0.01, *** *p* ≤ 0.001 (T-test). 50 µg/mL CuO are equal to 630 µM Cu^2+^.

Cellular copper uptake by CuO NP, CuO MP and CuCl_2_ was analyzed via graphite furnace atomic absorption spectroscopy (GF-AAS). As described in Materials and Methods, we applied a special post-incubation procedure, where the plasma membrane was removed to avoid any overestimation of intracellular copper levels due to an incomplete elimination of particles from the cellular surface, and thus quantified copper levels within the soluble cell fraction. Within our previous study, CuO NP were found to result in a pronounced dose-dependent intracellular copper overload up to millimolar concentrations, while CuCl_2_ showed the lowest and almost constant copper levels up to 400 µM independent from the applied dose. CuO MP resulted in intermediate but also concentration-dependently increased copper levels [8]. Within the present study, the intracellular copper content was investigated after 8 h incubation in the absence or presence of the dynamin-dependent endocytosis inhibitor OH-dyn (Figure 2A), as well as after 24 h in the absence or presence of bafA1 (Figure 2B). The shorter co-incubation time in case of OH-dyn was chosen due to its high cytotoxicity after 24 h treatment, as described above.

The basal copper concentration in BEAS-2B cells was found to be 20 µM. After 8 h incubation, treatment with CuO NP resulted in the highest intracellular copper levels, namely 950 µM, considerably higher as compared to the CuO MP (560 µM) or CuCl_2_ (360 µM). Co-treatment with OH-dyn diminished intracellular copper content by about 50% to 490 µM in the case of CuO NP, and—even more pronounced—by about 80% to 130 µM in case of CuO MP. No effect of OH-dyn was observed in the case of CuCl_2_, excluding any unspecific impact of the inhibitor on the homeostatic copper uptake. 

After 24 h incubation, intracellular copper accumulation was comparable to 8 h in case of CuO NP and CuO MP. However, higher copper concentrations, namely 770 µM, were observed in case of CuCl_2_. An impact of bafA1 was restricted to CuO NP; surprisingly, intracellular copper accumulation was markedly reduced by 60% from 1050 µM to 640 µM. In contrast, bafA1 did not alter intracellular copper levels in case of CuO MP and CuCl_2_.

### 3.4. Intracellular Glutathione (GSH) Level

**Figure 3:**
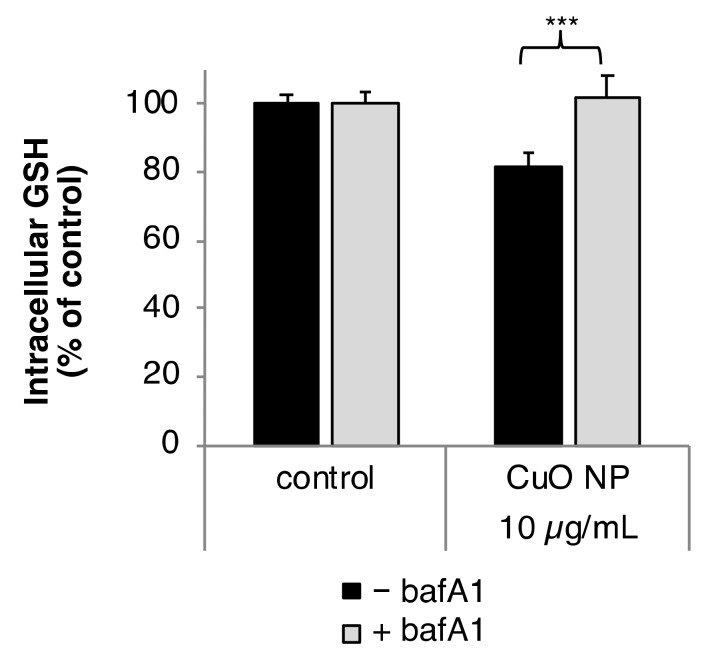
Impact of CuO NP on intracellular glutathione (GSH) level in the presence or absence of bafA1. BEAS-2B cells were treated CuO NP with or without bafA1 for 2 h. Shown are the mean values of five determinations derived from three independent experiments ± SD. Statistically significant different from CuO NP treatment: *** *p* ≤ 0.001 (T-test).

Modulation of intracellular GSH level by CuO NP depending on bafA1 was analyzed according to the method established by Tietze [24]. Intracellular GSH content was diminished to 80% of control after 2 h treatment with 10 µg/mL CuO. Co-treatment with bafA1 prevented the CuO NP-induced reduction of intracellular GSH completely (Figure 3). 

### 3.5. Gene Expression Analyses

Gene expression analyses via high-throughput qRT-PCR established previously in our group, quantifying the impact of 96 samples in parallel on the expression of 95 genes crucial for maintaining genomic stability. Selected genes were related to metal homeostasis, (oxidative) stress response as well as DNA repair, cell cycle regulation and apoptosis; a detailed description including a complete list of genes and respective proteins as well as their assignments to the respective groups has been published [13]. Applying this approach in a previous study in BEAS-2B cells after treatment with CuO NP, CuO MP as well as CuCl_2_, revealed a strong induction of copper uptake-related metallothionein genes, oxidative stress sensitive and inflammatory genes, anti-oxidative defense-associated genes, as well as the induction of cell cycle inhibitor gene *CDKN1A* (p21) and the pro-apoptotic *PMAIP1* (Noxa) and *TNFRSF10B* (DR5). Moreover, we observed a down-regulation of genes coding for important DNA repair proteins, along with the induction of DNA damage inducible genes. The extent of modulation occurred strictly dose-dependent in the case of the particulate CuO compounds, reflecting the increase in intracellular copper concentrations; effects were far most pronounced in case of CuO NP, whereas alterations in gene expression after treatment with CuCl_2_ were restricted to cytotoxic concentrations with disturbed copper homeostasis [8]. Following up on this investigation, within the present work, the impact of CuO NP and CuO MP on gene expression profiles, related to the DNA damage response in the absence and presence of OH-dyn or bafA1, was investigated after 8 h or 24 h, respectively. Affected genes, respective proteins, as well as an overview of their functions, are provided in Table 2. 

### 3.6. Impact of OH-dyn

**Figure 4:**
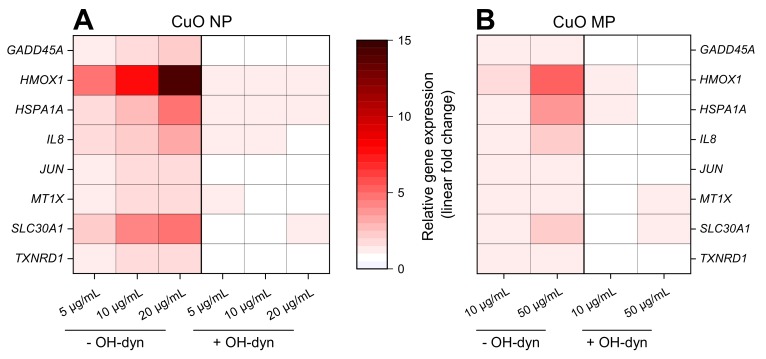
Impact of CuO NP (**A**) and CuO MP (**B**) on gene expression patterns related to copper homeostasis, (oxidative) stress, DNA damage and inflammation in the absence or presence of OH-dyn. BEAS-2B cells were treated with the different copper compounds with or without OH-dyn for 8 h. Shown are linear-fold changes of the relative gene expression in a heat map view from mean values of four determinations derived from two independent experiments (expression level of control = 1). Exact values including standard deviations are provided in supplementary Table S1.

8 h treatment with OH-dyn itself did already modulate the expression of some of the discussed genes, namely *HMOX1*, *HSPA1A* and *IL8*, albeit to a far lesser extent as compared to CuO NP exposure (Figure S1). Therefore, the effects of the co-treatments were related to the OH-dyn treated control. The impact of 8 h OH-dyn co-treatment on copper particle-modulated gene expression is summarized in a heat map view, displaying the relative gene expression related to the respective control (Figure 4). Strongest effects, starting at the lowest applied concentration of 5 µg/mL, were observed in case of CuO NP (Figure 4A). Most pronounced inductions were observed in case of the ROS-inducible *HMOX1* and *HSPA1A* genes. Furthermore, mRNA levels of the copper uptake-related genes *MT1X* and *SLC30A1* coding for metallothionein 1 and the zinc transporter ZnT-1, respectively, were induced by CuO NP. Also, transcription of the DNA damage inducible gene *GADD45A*, the pro-inflammatory gene *IL8*, the proto-oncogene *JUN* and the anti-oxidative defense-associated gene *TXNRD1* were enhanced in a strictly dose–dependent manner. In contrast, the modulation of expression occurred to a much lesser extent by CuO MP, with relevant, dose-dependent effects restricted to *HMOX1*, *HSPA1A*, *IL8* and *SLC30A1* transcript levels, even though up to 5 times higher copper amounts were applied (Figure 4B). Most strikingly, no alterations of gene expression compared to controls were observed after co-treatment with OH-dyn, neither in the case of CuO NP nor CuO MP. 

### 3.7. Impact of bafA1

**Figure 5:**
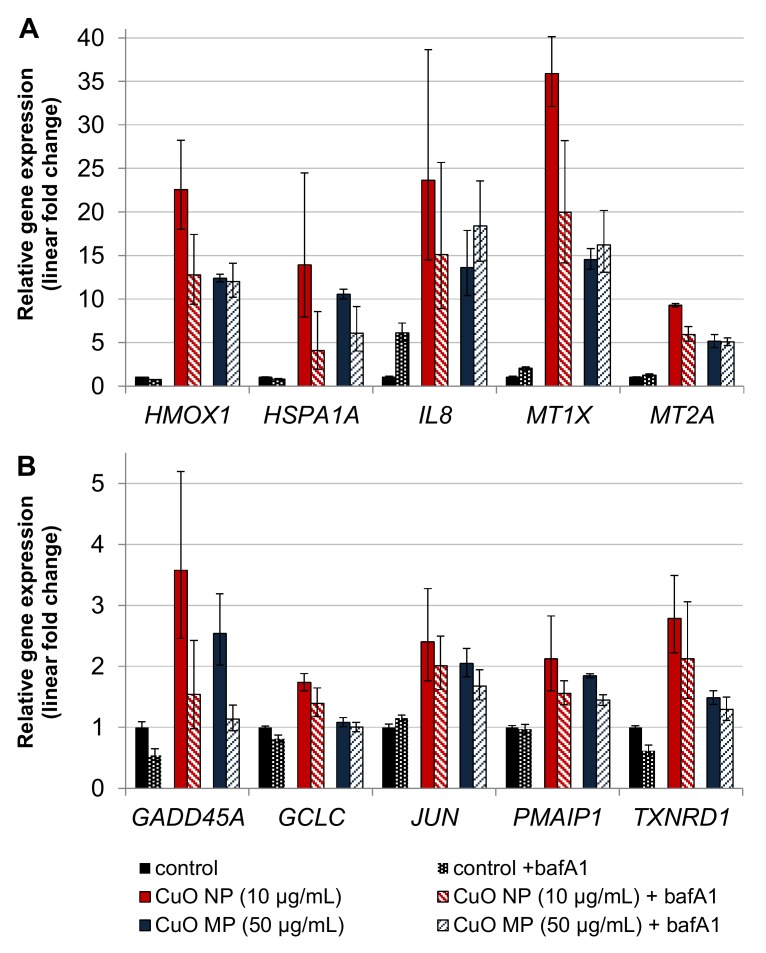
Impact of CuO NP and CuO MP on gene expression related to copper uptake, (oxidative) stress and inflammation (**A**) and DNA damage response, apoptosis, proliferation and anti-oxidative defense (**B**) in the presence or absence of bafA1. BEAS-2B cells were treated with the different copper compounds with or without bafA1 for 24 h. Shown are mean values of four determinations derived from two independent experiments ± SD.

Gene expression profiling applying 10 µg/mL CuO NP or 50 µg/mL CuO MP in the absence or presence of 24 h bafA1 co-treatment is shown in Figure 5A,B. Again, the most pronounced elevations of gene expression were observed in case of CuO NP when compared to CuO MP, even though the latter were applied at 5-fold higher amounts. BafA1 led to a strong, about 50% reduction of the CuO NP-mediated induction of the oxidative stress, as well as the copper uptake/homeostasis-related genes *HMOX1*, *HSPA1A*, *MT1X* and *MT2A*, as well as the DNA damage response gene *GADD45A*. In case of the pro-inflammatory *IL8* gene, results have to be interpreted with caution, due to the high standard deviations and the fact that a 6-fold transcriptional induction was already evident in case of bafA1 alone. Further inductions were observed in case of JUN, the pro-apoptotic *PMAIP1*, as well as the anti-oxidative associated genes *GCLC* and *TXNRD1*; in these cases, baf1A did not substantially alter the respective expression levels. In contrast to CuO NP, except for *HSPA1A* and *GADD45A*, bafA1 did not affect CuO MP-induced transcriptional changes. 

## 4. Discussion

Within the present study, the impact of the dynamin-dependent endocytosis inhibitor OH-dyn [19,20], and the vacuolar-type H^+^-ATPase inhibitor bafA1 attenuating lysosomal acidification [21,22] on cytotoxicity, cellular copper uptake, and especially the modulation of cellular signaling pathways involved in the maintenance of genomic stability by CuO NP, as compared to CuO MP, was investigated. 

As a prerequisite to estimate the impact of endocytosis, to quantify copper uptake and to discriminate between the cellular effects provoked by copper particle interactions at the outer cell membrane, and those derived from intracellular copper, a post-treatment protocol was applied. Within this procedure, the cells were lysed, and the membranes precipitated by centrifugation. Subsequently, the copper levels were quantified in the soluble cell fraction, excluding copper particles attached to the cell surface, which are not easily detached by washing procedures, thereby avoiding any overestimation of copper uptake. Intracellular copper concentrations were determined by AAS, and calculated based on the cell volume of BEAS 2B cells [8]; no discrimination between copper particles and copper ions is possible by this approach. 

In the absence of OH-dyn or bafA1, a pronounced copper accumulation up to 1 mM after 8 h or 24 h treatment with CuO NP was observed, while a lower increase in cellular copper was evident after incubation with CuO MP or CuCl_2_, confirming previous observations in this cell line [8]. In case of CuCl_2_, neither OH-dyn nor bafA1 affected the intracellular copper content, thus excluding any unspecific interactions between the inhibitors and copper uptake or homeostasis. 

On the contrary, OH-dyn caused a pronounced reduction of intracellular copper accumulation in the case of CuO NP and—even more pronounced—in the case of CuO MP, confirming their uptake via endocytosis. Indeed, dynamin-dependent endocytosis was identified as the main route for the internalization of the CuO NP, since a reduction of intracellular copper levels of about 50% was observed; considering the solubility of about 30% of the CuO NP in the cell culture medium (DMEM/FKS) within the 8 h time frame [7], at least part of the residual increase in intracellular copper is likely to result from extracellularly-released copper ions taken up via respective ion transporters. This assumption is also in agreement with the stronger inhibition of OH-dyn towards CuO MP uptake, since almost no solubility in the same cell culture medium was observed [7]. Nevertheless, a small impact of dynamin-independent endocytosis not inhibited by OH-dyn cannot be excluded [19,20]. 

In contrast to OH-dyn, an impact of the “lysosomal neutralizer” bafA1 was restricted to CuO NP, diminishing the amount of intracellular copper accumulation to about 60%. On the first sight, this observation is surprising, since this inhibitor would only be expected to inhibit solubilization and copper ion release in the lysosomal environment, and by applying AAS to quantify intracellular copper content, no discrimination between particulate and ionic copper is possible. However, if the lysosomes do not get acidified, the complete endocytic process is disturbed, and the internalized particles remain within the lysosomes, thereby preventing the recycling of protein or lipids essential for this process [28,29]. Consequently, endocytosis of the particles is slowed down and reduced during the incubation period of 24 h. The impact of the diminished release of redox-reactive copper ions by CuO NP in less acidic lysosomes became obvious when considering the other endpoints investigated within this study. Thus, co-treatment with bafA1 strongly reduced the cytotoxicity of CuO NP and prevented the CuO NP-induced GSH depletion after 2 h, indicating that oxidative stress occurs due to increased intracellular levels of copper ions. In support of this theory, an enhanced toxicity of CuO NP due to the increased intracellular solubility in an acidic environment resulting in elevated levels of copper ions, was also reported by other working groups [30,31]. In contrast to CuO NP, no impact of bafA1 was observed in case of CuO MP, in agreement with the low solubility of CuO MP in artificial lysosomal fluid (ALF), as compared to CuO NP published previously [7]. 

In the absence of either inhibitor, gene expression profiling after 8 h treatment with CuO NP revealed a strictly concentration-dependent induction of mainly so-called early response genes, namely *MT1X*, *SLC30A1*, *HMOX1*, *HSPA1A*, *GADD45A*, *IL8*, *JUN*, and *TXNRD1*, revealing elevated intracellular copper levels, the induction of oxidative stress, inflammation, DNA damage, inflammatory response, as well as an activation of the transcription factors AP-1 and MTF-1 [32,33,34,35,36,37,38]. A similar pattern was observed in case of CuO MP, however, far less pronounced. These observations highly corresponded to the previously published, detailed gene expression analyses with the same copper particles [8]. However, co-treatment with OH-dyn completely diminished the CuO NP- as well as CuO MP-induced gene inductions. This indicates that the observed changes in gene expression can be exclusively assigned to intracellular copper, with no impact of particle interactions with the outer membrane. 

Gene expression analyses after 24 h exposure to CuO particles revealed almost the same pattern as compared to the 8 h treatment; however, respective modulations were more pronounced. In the presence of bafA1, CuO NP-mediated gene inductions were reduced to a high degree by bafA1 in the case of the ROS-inducible *HMOX1* and *HSPA1A*, genotoxic stress-inducible *GADD45A*, pro- inflammatory *IL8*, as well as the copper uptake related *MT1X* and *MT2A*. Thus, with respect to the modulation of those stress responsive genes, the acidification of lysosomes, and in consequence the release of copper ions, were of major importance, signaling elevated levels of ROS, with the consequence of oxidative and genotoxic stress, as well as inflammatory effects [34,36,37,38,39]. These protective interactions in the presence of bafA1 correspond to the reduced intracellular copper accumulation, the prevented GSH-depletion and reduced cytotoxicity of CuO NP in the presence of bafA1 on the functional level. 

On the other hand, no impact of bafA1 was observed concerning the transcriptional modulation of the pro-apoptotic gene *PMAIP1*, the proto-oncogene coding *JUN* assigned to AP-1 activation [32] and the anti-oxidative genes *GCLC* and *TXNRD1* mainly induced by activated Nrf2 [40]. Thus, the amount of intracellular copper and ROS generation in the presence of bafA1 seemed still sufficient for the activation of these genes, which may again be explained by uptake of copper ions released extracellularly from the CuO NP, but also from low level intracellular copper ion release in the presence of bafA1. In case of CuO MP, except for *HSPA1A* and *GADD45A*, no impact of bafA1 on gene expression alterations was observed, in agreement with its low solubility, even in the acidic lysosomal environment. 

## 5. Conclusions

Taken together, our findings strongly support the leading role of the dynamin-dependent endocytosis and lysosomal acidification on CuO NP-induced cellular toxicity, provoking a distinct release of redox-active copper ions, and causing highly elevated intracellular copper levels. Toxicity profiles correspond to the induction of disturbed metal homeostasis, oxidative and genotoxic stress, as well as the activation of redox-sensitive transcription factors, thereby confirming previous investigations. They exclude the interactions of CuO NP with the outer cell membrane as contributing factors to their toxicity. In consequence, the results add strong support to the proposed Trojan horse-type mechanism accounting for CuO NP toxicity. Furthermore, they support the impact of particle size on CuO-induced cellular effects, also as a basis for toxicological risk assessment.

## Figures and Tables

**Table 1:** Characterization of the applied CuO particles (summarized from reference [7]).

Particles	Size Range (nm)	Primary Particle Size (nm)	SSA (m^2^/g)	Hydrodynamic Size (nm)	ζ-potential (mV)
CuO NP	20–200 (TEM)	55 *	17.23	(DMEM/FCS)	−13.1 (DMEM/FCS)
CuO MP	500–10,000 (TEM)	1289 *	0.74	nd **	nd **

TEM: transmission electron microscopy, SSA: surface specific area, * calculated based on SSA, ** not determined due to rapid sedimentation of the particles.

**Table 2:** Genes affected in a relevant manner by the CuO particles in the presence or absence of the inhibitors OH-dyn and/or bafA1.

Gene	Protein	Specific Function
*GADD 45A*	growth arrest and DNA-damage-inducible, alpha (GADD45A)	DNA damage signaling
*HMOX1*	heme oxygenase (decycling) 1 (HO1)	heme catabolism; oxidative stress response
*HSPA1A*	heat shock 70kDa protein 1A (hsp70)	chaperone; oxidative stress response
*IL8*	interleukin 8 (IL8)	chemokine; inflammatory response
*JUN*	jun proto-oncogene (c-jun)	part of the early response transcription factor AP-1, cell proliferation
*MT1X, MT2A*	metallothionein 1X (MT1X), metallothionein 2A (MT2A)	metal homeostasis
*SLC30A1*	solute carrier family 30 (zinc transporter), member 1 (ZnT1)	transcription factor, metal homeostasis
*TXNRD1*	thioredoxin reductase 1 (TxrR)	selenium metabolism; oxidative stress response
*GCLC*	glutamate-cysteine ligase, catalytic subunit (GCL)	GSH synthesis, oxidative stress response
*PMAIP1*	phorbol-12-myristate-13-acetate-induced protein 1 (Noxa)	pro-apoptotic gene, member of the bcl-2 family

## Supplementary Materials

The following are available online at https://www.mdpi.com/2079-4991/10/4/679/s1, Figure S1: Supporting information on the impact of OH-dyn on the expression of genes modulated by the copper compounds, Table S1: supporting information on the impact of CuO NP (A) and CuO MP (B) on gene expression levels related to copper homeostasis, (oxidative) stress, DNA damage and inflammation in the absence or presence of OH-dyn. BEAS-2B cells were treated with the different copper compounds with or without OH-dyn for 8 h.

## Abbreviations

AAF: Artificial alveolar fluid; ALF: Artificial lysosomal fluid; AP-1: activator protein 1; bafA1: bafilomycin A1; BET: Brunauer–Emmett–Teller analysis; CDKN1A: cyclin-dependent kinase inhibitor 1A; Cq: Quantitation cycle; CuCl_2_: Copper chloride, CuO MP: Copper oxide microparticles; CuO NP: Copper oxide nanoparticles, DLS: Dynamic light scattering; DR5: death receptor 5; EDX: Energy-dispersive X-ray spectroscopy; GADD45A: growth arrest and DNA-damage-inducible, alpha; GF-AAS: Graphite furnace atomic absorption spectrometry; GSH: Glutathione; hCTR1: high-affinity copper transporter 1; HMOX1: heme oxygenase; HSPA1A: heat shock 70kDa protein 1A; ICP-MS: Inductively coupled plasma mass spectrometry; IL8: interleukine 8; MT1X: metallothionein 1X; MT2A: metallothionein 2A; MTF-1: Metal-regulatory transcription factor 1; Nrf2: Nuclear factor (erythroid-derived 2)-like 2; OH-dyn: hydroxyl-dynasore; PMAIP1: phorbol-12-myristate-13-acetate-induced protein 1; ROS: Reactive oxygen species; RT-qPCR: Reverse transcription quantitative polymerase chain reaction; SCL30A: solute carrier family 30 (zinc transporter), member 1; SEM: Scanning electron microscopy; SOD: Superoxide dismutase; TEM: Transmission electron microscopy; TNFRSF10B: tumor necrosis factor receptor superfamily, member 10b; TXNRD1: thioredoxin reductase 1;.V-ATPase: vacuolar type H^+^-ATPase; ZP: Zeta potential; ZnT-1: zinc transporter 1
