# Supplementary material for: Impact of Endocytosis and Lysosomal Acidification on the Toxicity of Copper Oxide Nano- and Microsized Particles: Uptake and Gene Expression Related to Oxidative Stress and the DNA Damage Response
Source: Nanomaterials (Basel). 2020 Apr 3;10(4):679. doi: 10.3390/nano10040679 (PMC7221514; doi:10.3390/nano10040679)
Supplement: Supplementary file 1 [file nanomaterials-10-00679-s001.pdf]

nanomaterials

Supporting information on the impact of OH-dyn on the expression of genes modulated by the copper compounds in BEAS-2B cells.

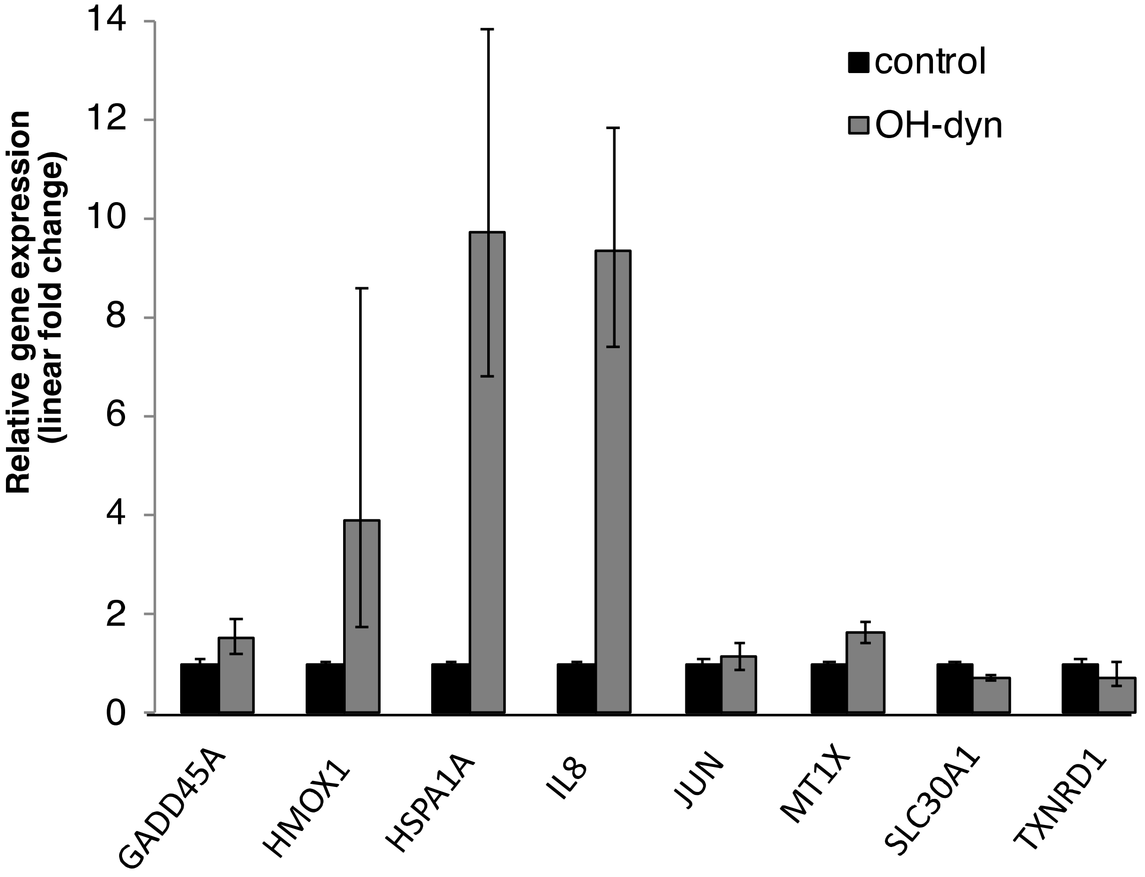

Figure S1. BEAS-2B cells were treated with the different copper compounds and OH-dyn for 8 h. Shown are mean values of four determinations derived from two independent experiments ± SD.

Table 1. Impact of CuO NP (A) and CuO MP (B) on gene expression levels related to copper homeostasis, (oxidative) stress, DNA damage and inflammation in the absence or presence of OH-dyn. BEAS-2B cells were treated with the different copper compounds with or without OH-dyn for 8 h. Shown are mean values of four determinations derived from two independent experiments +/- SD.

A:CuO NP

**- OH-Dyn**	**control**			**CuO NP 5 µg/mL**			**CuO NP 10 µg/mL**			**CuO NP 20 µg/mL**		
**Gene**	**Mean**	SD err+	SD err-	**Mean**	SD err+	SD err-	**Mean**	SD err+	SD err-	**Mean**	SD err+	SD err-
GADD45A	**1.00**	0.09	0.08	**1.40**	0.33	0.27	**1.83**	0.84	0.58	**2.20**	0.87	0.63
HMOX1	**1.00**	0.06	0.05	**4.84**	1.48	1.14	**7.89**	6.62	3.60	**14.44**	8.61	5.39
HSPA1A	**1.00**	0.04	0.03	**1.80**	0.43	0.35	**2.74**	1.78	1.08	**4.98**	3.13	1.92
IL8	**1.00**	0.05	0.04	**1.64**	0.17	0.15	**2.10**	0.83	0.60	**3.42**	1.68	1.13
JUN	**1.00**	0.09	0.08	**1.20**	0.19	0.17	**1.79**	0.26	0.23	**1.79**	0.36	0.30
MT1X	**1.00**	0.06	0.06	**1.33**	0.13	0.12	**1.67**	0.12	0.11	**1.92**	0.30	0.26
SLC30A1	**1.00**	0.03	0.03	**2.44**	0.18	0.17	**4.29**	1.18	0.93	**4.89**	0.58	0.52
TXNRD1	**1.00**	0.10	0.09	**1.34**	0.24	0.20	**1.85**	0.13	0.12	**1.84**	0.06	0.06

**+ OH-Dyn**	**control**			**CuO NP 5 µg/mL**			**CuO NP 10 µg/mL**			**CuO NP 20 µg/mL**		
**Gene**	**Mean**	SD err+	SD err-	**Mean**	SD err+	SD err-	**Mean**	SD err+	SD err-	**Mean**	SD err+	SD err-
GADD45A	**1.00**	0.14	0.12	**0.93**	0.17	0.15	**0.98**	0.29	0.22	**0.95**	0.08	0.07
HMOX1	**1.00**	0.06	0.06	**1.01**	0.38	0.27	**1.07**	0.19	0.16	**1.05**	0.22	0.18
HSPA1A	**1.00**	0.17	0.14	**1.29**	0.50	0.36	**1.01**	0.62	0.39	**1.10**	0.08	0.08
IL8	**1.00**	0.02	0.02	**1.01**	0.06	0.05	**1.01**	0.11	0.10	**0.95**	0.10	0.09
JUN	**1.00**	0.03	0.02	**0.94**	0.07	0.06	**0.81**	0.08	0.07	**0.94**	0.09	0.08
MT1X	**1.00**	0.05	0.05	**1.29**	0.47	0.35	**0.93**	0.02	0.02	**0.98**	0.05	0.05
SLC30A1	**1.00**	0.07	0.07	**0.96**	0.23	0.18	**0.95**	0.21	0.17	**1.14**	0.07	0.07
TXNRD1	**1.00**	0.06	0.06	**0.92**	0.16	0.14	**0.81**	0.15	0.13	**1.00**	0.19	0.16

B: CuO MP

- OH-Dyn	control			CuO MP 10 µg/mL			CuO MP 50 µg/mL		
**Gene**	**Mean**	SD err+	SD err-	**Mean**	SD err+	SD err-	**Mean**	SD err+	SD err-
GADD45A	**1.00**	0.12	0.10	**1.31**	0.17	0.15	**1.45**	0.20	0.17
HMOX1	**1.00**	0.04	0.04	**1.70**	0.21	0.19	**5.18**	0.66	0.59
HSPA1A	**1.00**	0.03	0.03	**1.48**	0.14	0.13	**3.93**	0.56	0.49
IL8	**1.00**	0.05	0.05	**1.16**	0.10	0.09	**2.12**	0.44	0.36
JUN	**1.00**	0.05	0.05	**1.08**	0.10	0.09	**1.12**	0.08	0.07
MT1X	**1.00**	0.08	0.08	**1.21**	0.03	0.03	**1.35**	0.08	0.08
SLC30A1	**1.00**	0.02	0.02	**1.46**	0.06	0.06	**2.34**	0.12	0.11
TXNRD1	**1.00**	0.03	0.03	**1.27**	0.03	0.03	**1.26**	0.10	0.09

+ OH-Dyn	control			CuO MP 10 µg/mL			CuO MP 50 µg/mL		
**Gene**	**Mean**	SD err+	SD err-	**Mean**	SD err+	SD err-	**Mean**	SD err+	SD err-
GADD45A	**1.00**	0.04	0.03	**0.89**	0.12	0.10	**0.89**	0.04	0.04
HMOX1	**1.00**	0.05	0.05	**1.08**	0.19	0.16	**0.66**	0.16	0.13
HSPA1A	**1.00**	0.10	0.09	**1.13**	0.18	0.16	**0.69**	0.14	0.12
IL8	**1.00**	0.03	0.03	**0.95**	0.15	0.13	**0.89**	0.11	0.10
JUN	**1.00**	0.02	0.02	**0.90**	0.09	0.08	**0.99**	0.16	0.14
MT1X	**1.00**	0.03	0.03	**0.87**	0.04	0.04	**1.03**	0.07	0.06
SLC30A1	**1.00**	0.03	0.03	**0.88**	0.05	0.05	**1.04**	0.03	0.03
TXNRD1	**1.00**	0.06	0.05	**0.84**	0.09	0.08	**0.69**	0.08	0.08
